# Medical workforce in the United States

**DOI:** 10.1002/acm2.13799

**Published:** 2022-11-15

**Authors:** Edward I. Bluth, Donald P. Frush, M. Elizabeth Oates, Jeanne LaBerge, Hubert Y. Pan, Wayne D. Newhauser, Seth A. Rosenthal

**Affiliations:** ^1^ Department of Radiology Ochsner Clinic Foundation New Orleans Louisiana USA; ^2^ Department of Radiology Duke University Durham North Carolina USA; ^3^ Department of Radiology University of Kentucky College of Medicine Lexington Kentucky USA; ^4^ Department of Radiology University of California San Francisco California USA; ^5^ Sutter Radiation Oncology Center Sacramento California USA; ^6^ Department of Physics and Astronomy Louisiana State University and Mary Bird Perkins Cancer Center Baton Rouge Louisiana USA; ^7^ Sutter Radiation Oncology Center Roseville California USA

**Keywords:** medicine, nuclear medicine/interventional radiology, oncology/nuclear radiology, physician, radiology/diagnostic, radiology/radiation, workforce

## Abstract

This section focuses on the professional workforce comprised of the primary medical specialties that utilize ionizing radiation in their practices. Those discussed include the specialties of radiology and radiation oncology, as well as the subspecialties of radiology, namely diagnostic radiology, interventional radiology, nuclear radiology, and nuclear medicine. These professionals provide essential health care services, for example, the interpretation of imaging studies, the provision of interventional procedures, radionuclide therapeutic treatments, and radiation therapy. In addition, they may be called on to function as part of a radiologic emergency response team to care for potentially exposed persons following radiation events, for example, detonation of a nuclear weapon, nuclear power plant accidents, and transportation incidents. For these reasons, maintenance of an adequate workforce in each of these professions is essential to meeting the nation's future needs. Currently, there is a shortage for all physicians in the medical radiology workforce.

## 4.1 INTRODUCTION

This section focuses on the professional workforce comprised of the primary medical specialties that utilize ionizing radiation in their practices. Those discussed include the specialties of radiology and radiation oncology, as well as the subspecialties of radiology, namely diagnostic radiology, interventional radiology, nuclear radiology, and nuclear medicine. These professionals provide essential health care services, for example, the interpretation of imaging studies, the provision of interventional procedures, radionuclide therapeutic treatments, and radiation therapy. In addition, they may be called on to function as part of a radiologic emergency response team to care for potentially exposed persons following radiation events, for example, detonation of a nuclear weapon, nuclear power plant accidents, and transportation incidents. For these reasons, maintenance of an adequate workforce in each of these professions is essential to meeting the nation's future needs. Of note, other medical specialties that use ionizing radiation, such as cardiology, orthopedics, and urology, are considered outside the scope of this report.

## 4.2 DEFINITIONS OF THE MEDICAL RADIATION PROFESSIONS

### 4.2.1 Radiology

The term “radiology” encompasses the scientific fields that utilize high‐energy radiation for imaging purposes. All specialists working within these fields are medical doctors or doctors of osteopathic medicine who have received extensive training in the application of various radiological modalities for clinical diagnosis and therapeutic interventions. With respect to the specialties and subspecialties highlighted in this report, diagnostic radiologists are physicians who specialize in the application, supervision, and interpretation of noninvasive imaging techniques to diagnose disease processes and injuries affecting patients. The methodologies that make use of ionizing radiation include radiography and fluoroscopy (X‐rays) and computed tomography (CT) scans; nonionizing methodologies include imaging with ultrasound and magnetic resonance imaging.

Of the various subspecialties, interventional radiologists focus on the diagnostic and therapeutic aspects of patient care and have recognized expertise in diagnostic imaging and image‐guided procedural treatments. These radiologists use diagnostic imaging tools, such as fluoroscopy, CT, and ultrasound to perform a wide range of therapeutic procedures, such as dilating a narrowed blood vessel (angioplasty) or draining an obstructed kidney (nephrostomy). Nuclear radiology is a well‐established subspecialty of diagnostic radiology; nuclear radiologists are trained to practice the full scope of interrelated multimodality diagnostic radiological imaging and radiopharmaceutical‐related imaging.^1^ The field of nuclear medicine is an independent, but related, discipline, although their scope of practice is generally limited to radiopharmaceutical‐related imaging. Both nuclear radiology and nuclear medicine employ diagnostic radiopharmaceuticals (“tracers”) for the purpose of imaging a wide variety of benign and malignant disease processes; they also prescribe therapeutic radiopharmaceuticals to treat certain conditions. Inherent in all of these fields is the need to understand and manage the potential health risks and safety requirements of both patients and staff. Included among these risks are musculoskeletal injuries, radiation injuries, and needle sticks.^2^, ^3^, ^4^

### 4.2.2 Radiation oncology

Radiation oncologists are physicians who specialize in the utilization of ionizing radiation for medical treatment. This is delivered primarily for malignant conditions, with approximately 50% of cancer patients receiving radiation therapy as part of their treatment.^5^ Radiation is used also for a wide variety of benign and life‐threatening diseases, such as arteriovenous malformations of the brain. Radiation oncologists often work in conjunction with medical and surgical oncology colleagues. They decide the treatment protocol (radiation modality and dose) and evaluate and approve the treatments plans (radiation field distribution). They are also responsible for explaining the risks, benefits, and rationale of radiation treatment to patients and managing the side effects of treatment. Radiation oncologists are subsequently involved in the follow‐up and survivorship care of patients who have undergone treatment with radiation therapy.

## 4.3 GENERAL CHARACTERISTICS OF THE WORKFORCES

Radiation professionals comprise small, but essential, components of the medical workforce. For example, radiologists represent approximately 3.1% of the 892 million physicians practicing in 2017.^6^ However, their clinical influence affects the diagnosis and treatment of virtually all patients seen by practicing physicians. In addition, of the approximately 23 000 practicing oncologists in the US in 2016, roughly 4000 were radiation oncologists.^7^, ^8^ Given that 50% of cancer patients will receive radiation as part of their treatment, radiation oncologists cover a large proportion of the services in this sector.^9^

### 4.3.1 Radiology

The characteristics of the radiology workforce reflect primarily those in diagnostic radiology, although data on the interventional and nuclear radiology and nuclear medicine sectors are included where available. The composition of the radiology workforce has shown some variability in recent years, with the percentage of the workforce defined as general radiologists dropping sharply from 35.2 % in 2012 to 10.4 % in 2017, although none of the other subspecialties changed significantly over the same period. Importantly, with the advent of the picture archiving and communication system and the electronic transfer of images, the interpretation of many components of diagnostic radiology, nuclear radiology, and nuclear medicine images can be made at a distance, allowing coverage of remote rural areas to become more feasible. This contrasts with some subspecialties of diagnostic radiology such as breast imaging, pediatrics, and ultrasound, as well as interventional radiology (and radiation oncology), which continue to require a physical presence.^10^, ^11^

#### 4.3.1.1 Diagnostic radiology

**FIGURE 1:**
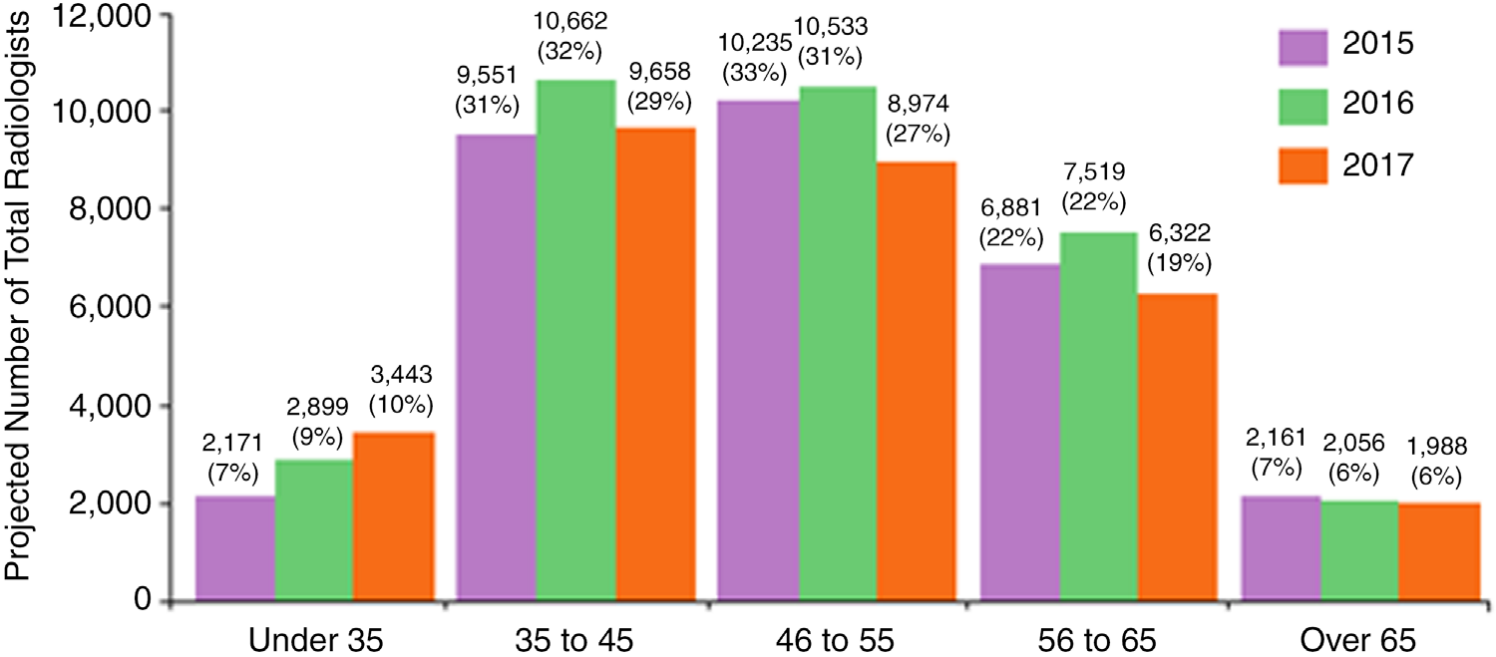
Estimated number of the total population of practicing radiologists by age, for the years 2015–2017, as identified by the 2017 American College of Radiology (ACR) Workforce Survey^12^, ^13^, ^14^

The diagnostic radiology workforce is currently made up of approximately 34 000 radiologists,^12^ with an additional >2100 mid‐level practitioners helping perform diagnostic examinations and over 250 000 radiologic technologists participating in the process of image generation. The distribution in age of practicing radiologists has remained relatively stable since 2012, with the bulk of the workforce (∼60%) being between 35 and 55 years. Ten percent of the radiologists working in 2017 (*n* = 3433) were under the age of 35; 19% (*n* = 8332) 56–65 years and 6% (*n* = 1988) >65 years (Figure 1).^12^, ^13^, ^14^ Follow‐up information has been collected in the American College of Radiology (ACR) 2018 and 2019 surveys with the resultant data remaining very similar.^15^, ^16^ In the same year, 25% of practices had a radiologist retiring, although 24% of the groups also reported having a retired radiologist continuing to be employed in some capacity.

A number of factors may affect the characteristics and needs of the future radiology workforce. For example, in 2017, 84% of all radiologists worked full‐time, with only 16% working part‐time. However, there was a statistically significant difference between the numbers of women (30%) and men (10%) working part‐time (*p* < 0.01), with only 21.5% of women older than 45 working full‐time. If the number of women in radiology residency training programs increases, and the trend of increasing part‐time work continues, the actual number of radiology training slots may need to be increased to maintain adequate patient access in the future.

#### 4.3.1.2 Interventional radiology

Interventional radiology was initially recognized in 1994 as a subspecialty of diagnostic radiology and was then named “vascular and interventional radiology” (VIR). More recently, the American Board of Medical Specialties (ABMS) recognized interventional radiology as a unique specialty in 2012, and an associated residency program was created in 2014. Since the first class of residents in this specialty will not graduate until 2023, the characteristics of the interventional radiology workforce are projected from those known of the VIR.

In 2017, there were 2700 board‐certified subspecialists in VIR in the US. In 2017, there were 3450 physician members of the Society of Interventional Radiology (SIR), so that the actual number of radiologists who perform IR procedures may be somewhat higher. Indeed, some basic IR procedures may be performed by diagnostic radiologists who receive training in basic IR procedures during residency. In many groups, multispecialty radiologists may help groups to help cover basic interventional procedures.^17^ The 2017 ACR workforce survey determined that there are 4318 radiologists currently employed who are identified as general interventionalists.^12^ The distribution of IRs is not well documented, nonetheless there may not be a great need for interventional radiologists in rural areas; it is a highly specialized field and is most commonly practiced at tertiary or quaternary referral centers or academic medical centers.

#### 4.3.1.3 Nuclear radiology and nuclear medicine

**FIGURE 2:**
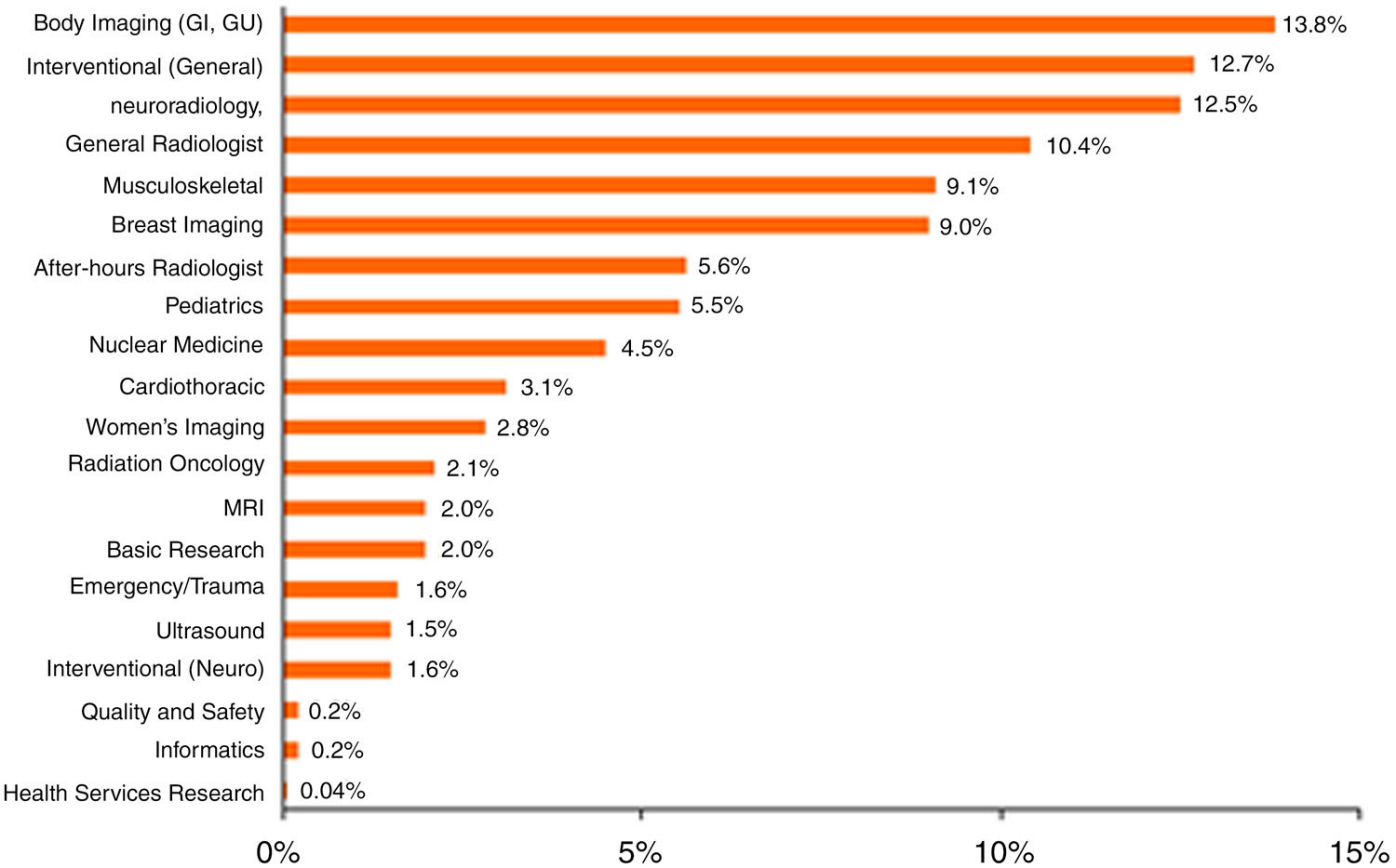
Percentage of radiologists and radiation oncologists currently employed by subspecialty type in 2016 as identified by the 2017 American College of Radiology (ACR) Workforce Survey^12^

Readily available and accurate workforce data on nuclear radiologists and nuclear medicine physicians are lacking. According to the ACR, nuclear practitioners represent approximately 4.5% of the workforce; however, these data do not differentiate between radiologists and nonradiologists^12^ (Figure 2). Since 1976, The American Board of Radiology (ABR) has issued >375 nuclear radiology subspecialty certifications to board‐certified diagnostic radiologists. Since July 2017, when the redesigned ABR 16‐month pathway within the 48‐month diagnostic radiology residency went into effect, there has been renewed interest in nuclear radiology by diagnostic radiology residents still in training.

Since 1972, The American Board of Nuclear Medicine (ABNM) has certified >5800 diplomates; however, not all are currently in practice nor are located in the USA. The ABNM has 3877 diplomates with lifetime certificates who were certified between 1972 and 1991; it is not known how many of these diplomates remain in the workforce. In contrast, the ABNM has 1868 diplomates with time‐limited certificates who were initially certified between 1992 and 2017; most are still in the workforce, although the exact number is not known. There are >3600 diplomates with lifetime or time‐limited certificates who are actively participating in maintenance of certification (MOC), most of whom practice in the USA; however, there are more diplomates in practice than those who participate in MOC. The total number of ABNM diplomates who were certified at one time by the ABR is 3021 (53%), thus more than half of the ABNM‐certified practitioners are diagnostic radiologists who pursued nuclear medicine training, either before or after diagnostic radiology training. Regarding the workforce pipeline, there are currently 41 the Accreditation Council for Graduate Medical Education (ACGME)‐accredited Nuclear Medicine residency programs with a total of 154 approved positions.

### 4.3.2 Radiation oncology

The following data are based on an ASTRO workforce study conducted in 2017.^18^ At that time, there were approximately 3700 practicing radiation oncologists in the United States, with a median age of 51 years and a male‐to‐female ratio of 2.9–1 (∼70% male). The majority of radiation oncologists (58%) work in a hospital‐setting, with a large minority in private practice (38%). Radiation oncologists see a median number of 250 new patient consults per year and have an average of 20 patients on treatment each week. The overall number of practices is the highest in the South (33.8%), followed by the Midwest (24.7%), West (21.9%), and Northeast (19.7%); however, there is an overall lack of geographic diversity, with the greatest number of radiation oncologists found in resource‐rich suburban (41%) or urban (47%) settings rather than rural practices (13%). This geographic distribution also reflects geographic disparity with respect to patient care, with the more modern modalities, such as stereotactic body radiation therapy (SBRT) and stereotactic radiosurgery (SRS), having utilization rates 30% lower in rural areas than those found in urban practices.

In the 2017 survey,^18^ respondents were more likely to express fears of an oversupply of radiation oncology practitioners, rather than a shortage. This may have reflected perceived changes in practice, for example, the adoption of more hypofractionated protocols, the relatively low number of proposed retirees within the next 5 years (∼735) versus residents potentially entering the workforce (∼950), and the preferential bias toward working within urban settings, since there is a perennial shortage of available positions in desirable areas.

## 4.4 EDUCATION AND TRAINING PATHWAYS

Requirements for the education and training of radiation medical professionals are described below, with the undergraduate and graduate pathways being dictated by those necessary for a medical degree and are, therefore, the same for diagnostic radiology, interventional radiology, nuclear radiology and nuclear medicine, and radiation oncology.

### 4.4.1 Organizations involved in education

The primary organizations involved in the postgraduate training and education of the medical radiation fields include the Accreditation Council for Graduate Medical Education (ACGME), which provides accreditation and review of graduate level (internship and residency) training programs. The main organization focusing on medical student training in radiation specialties is the Alliance of Medical Student Educators in Radiology and, for postgraduate medical education, the organizations include the Association of Program Directors in Radiology (APDR), the Association of Program Directors in Interventional Radiology, the Nuclear Medicine Program Directors Association, and the Association for Directors of Radiation Oncology Programs (ADROP). In addition, related professional societies offer further education and training opportunities.

### 4.4.2 Undergraduate education

A requirement for entrance into medical school in the United States is a 4‐year undergraduate education. Included in the requirements for admission to most medical schools are courses with laboratory experience in biology, general chemistry, organic chemistry, biochemistry, and physics.

### 4.4.3 Graduate education

Medical education in the United States is typically 4 years. There are a few combined programs for undergraduate education and medical training that occur over a shorter period of time (e.g., 6 years) rather than the traditional 4 years for each. In addition, graduate training can be embedded within the medical school training period, such as that resulting in a PhD or master's degrees, extending the overall time until a medical degree is conferred. Medical training is a combination of classwork, that is, didactic sessions on sciences related to medical practice in health and disease, and clinical experience. The latter involves working with residents, faculty, and other health care experts, in both in‐ and outpatient settings, in a supervised fashion traditionally covering surgical, medical, pediatric, community medicine, and other major specialties. The final, usually fourth, year is typically spent on elective clinical services, doing subinternships or acting internships (more intensive clinical rotations), and interviewing for residency positions.

### 4.4.4 Postgraduate education

In medicine, postgraduate education is commonly referred to as Graduate Medical Education (GME), which refers to formal medical education pursued after receipt of the MD or DO degree, including internship, residency, and subspecialty fellowship programs. GME leads to eligibility for state licensure and board certification.

#### 4.4.4.1 Radiology

Radiology is one of a small number of medical specialties that requires a year of initial training, designated as the internship or clinical year. Internships are classified as categorical (internship and residency within a single institution), preliminary, or transitional.^19^, ^20^ The preliminary clinical year is typically in the domain of surgery or internal medicine; a transitional clinical year experience is often varied by curricula and responsibilities. After the preparatory clinical year, the individual enters into a residency for specialized training.

#### 4.4.4.2 Diagnostic radiology

Diagnostic radiology residency, in general, is *4* years, and the vast majority (>90%) of radiology residents subsequently undertake one or more fellowships for subspecialty training.^19^ Most of these are 1 year in length, although some, such as pediatric or neuroradiology, have options for 2 years of training. In diagnostic radiology, fellowship status is conferred through one of two pathways, either through an ACGME‐accredited fellowship (abdominal radiology, musculoskeletal radiology, nuclear radiology, and pediatric radiology) or via a nonaccredited path that includes training in areas such as breast imaging, women's imaging (a combination including breast, abdominal, and pelvic imaging), emergency radiology, pediatric interventional and neuroradiology, and magnetic resonance imaging, among others.^19^ In this second pathway, fellowship status results through the designated program director confirming successful completion of the institutionally established program requirements for the fellowship. In general, these two pathways are different ways to arrive at subspecialty expertise and are not differentiated in terms of higher or lower value.

#### 4.4.4.3 Interventional radiology

In 2014, the ACGME agreed to create a new residency program in interventional radiology.^21^ The American Board of Radiology (ABR) issued its first certificates in the specialty in 2017. The new training program in interventional radiology residency consists of two options: a discipline‐specific integrated 5‐year program or an independent 2‐year residency for graduates from a diagnostic radiology residency (Laberge and Anderson, 2015). To date, there are approximately 60 accredited integrated interventional radiology residency programs offering >120 positions, and ACGME anticipates an additional 30 programs will apply for the independent residency, offering approximately 100–150 additional positions.

#### 4.4.4.4 Nuclear radiology and nuclear medicine

Diagnostic radiologists may subspecialize in nuclear radiology through one of several training pathways available in an ACGME‐accredited diagnostic radiology residency and nuclear radiology fellowship program, or a nuclear medicine residency.^22^, ^23^ After a clinical year, these pathways are four or 5 years in length. In addition, nonradiologists may specialize in nuclear medicine by completing residency training in a 3‐year ACGME‐accredited nuclear medicine program.^23^

#### 4.4.4.5 Radiation oncology

Approximately 200 radiation oncology resident positions are available each year through the National Residency Matching Program for a total of 5 years of training.^24^ ACGME requirements for radiation oncology programs include instruction in radiation physics, cancer and radiation biology, and safety.^25^

### 4.4.5 On‐the‐job training

There is no option for on‐the‐job training for any of the described fields in the United States.

### 4.4.6 Professional certification and licensure

Board certification for radiologists and radiation oncologists is achieved almost entirely through the American Board of Radiology. The ABR, founded in 1934, is a not‐for‐profit organization and one of the 24 members of the ABMS.^26^ The ABR provides the requirements for initial board certification^27^ and ongoing maintenance of certification^28^ for all radiation oncologists.

#### 4.4.6.1 Radiology

Initial certification requirements include a qualifying core examination, which assesses performance in radiation modalities (including computed tomography, nuclear radiology, fluoroscopy, and radiography) and nonradiation modalities (magnetic resonance imaging and ultrasound, organ systems), medical physics (including performance pertaining to radiation use), as well as safety and quality, including components of radiation safety. Diagnostic radiologists will typically qualify for Authorized User (AU) status through the NRC or Agreement State for diagnostic radioactive materials and oral radioiodine therapies.

Nuclear radiologists are initially certified in diagnostic radiology and, thereafter, seek subspecialty certification in nuclear radiology through the ABR.^23^ They may also be certified in nuclear medicine by the ABNM,^29^ the specific certification process for nuclear medicine physicians who are not eligible for ABR certification. In addition, on the basis of their training and experience, nuclear radiologists and nuclear medicine physicians will typically be able to secure AU status for the full scope of diagnostic and therapeutic radioactive materials for human use,^1^, ^30^ encompassing the use of oral radioiodine, as well as parenteral alpha emitters, beta emitters, and photon‐emitting radionuclides with a photon energy below 150 keV.

Until recently, interventional radiologists held a primary certificate in diagnostic radiology and a subspecialty certificate in VIR. However, since October 2017, interventional radiologists who hold a VIR subspecialty certificate have transitioned to the new dual interventional radiology and diagnostic radiology certificate. Additional ABR subspecialty certification for both pediatric radiology and neuroradiology exists, with program oversight through ACGME.

#### 4.4.6.2 Radiation oncology

Initial certification of radiation oncologists includes a computer‐based qualifying examination with three parts: (1) medical physics for radiation oncology, (2) radiation and cancer biology; and (3) clinical radiation oncology. The qualifying examination encompasses all aspects of general oncology and radiation oncology, including treatment‐planning techniques, physics, radiation and cancer biology, and specific aspects from all elements of multimodality cancer care. Following successful completion of the qualifying examination, the candidate may advance to the Certifying (Oral) Examination. Certification in radiation oncology will typically confer eligibility to secure AU status for the full scope of therapeutic radioactive materials for human use.^1^, ^30^ These therapies encompass oral radioiodine, as well as parenteral, intra‐cavitary, and intra‐articular, alpha, beta, and photon‐emitting radionuclides.

### 4.4.7 Continuing education

In the context of the medical radiation workforce, continuing education (continuous medical education, [CME]) is associated with maintaining professional certification and licensure to practice medicine. CME supports continuous quality improvement, professional development, and quality patient care. In recent decades, the mandated time being spent on CME activities per individual has increased due to the goal of maintaining physician efficacy in a rapidly changing medical environment. It is not clear, with the changes in communication methodologies, such as web‐ and pod‐casts, and the dramatic increase in remote learning developed during the COVID‐19 pandemic, if the amount of time spent in obtaining CME credits will change over time and thereby significantly alter the capacity of the workforce. In addition, MOC guidelines have been established for diagnostic radiologists, interventional radiologists, and radiation oncologists by the American Board of Radiology.^28^ Some hospitals require licensure and often have credentialing requirements that depend on maintaining a certain amount of CME, another component of MOC.

## 4.5 PROFESSIONAL ASPECTS OF RELEVANCE TO WORKFORCE SUPPLY

### 4.5.1 Professional organizations

Radiologists engage with many national and international professional organizations and societies, many of which offer some level of training and education. The exact number is unclear, although the ACR, the Radiological Society of North America (RSNA), and the American Roentgen Ray Society are three of the largest domestic organizations. In addition, there are many subspecialty organizations that deal with, for example, organ systems, modalities, disorders, and scope of practice (e.g., emergency radiology organizations). The Society of Nuclear Medicine and Molecular Imaging (SNMMI) is a primary resource for nuclear radiology and nuclear medicine practitioners. The main professional society associated specifically with interventional radiology is the SIR with >8000 members, of whom approximately 3500 are physicians. Radiation oncologists participate in a large number of organizations, including the ASTRO, the American Radium Society, the Association of Residents in Radiation Oncology, the AACR, the American College of Radiology, the American Society of Clinical Oncology, the RSNA, and the American College of Radiation Oncology.

### 4.5.2 Interdependencies with other radiation professions

The major ABR disciplines of diagnostic radiology, interventional radiology, radiation oncology, and medical physics have, in combination, the longest, broadest, and deepest requirements for radiation through their use in diagnostic, therapeutic, and image‐guided procedures. Among these four disciplines are networks that extend into each discipline's community, with harmonization through the ABR with respect to board certification, maintenance of certification, and related educational domains. This affords effective multidirectional dialogues related to practice, education and other scholarly activities related to training and certification.

#### 4.5.2.1 Radiology

Radiologists work collaboratively with multiple professions, not only with other radiology subspecialties, but also with medical and health physicists, nuclear medicine physicians, nuclear pharmacists, nuclear medicine technologists, and radiation oncologists. It is also worth noting that ACGME program requirements for interventional radiology require a dedicated faculty member with expertise in medical physics, and, furthermore, that didactic instruction includes diagnostic radiologic physics, instrumentation, and radiation biology, as well as patient and medical personnel safety (i.e., radiation protection).

#### 4.5.2.2 Radiation oncology

Radiation oncologists work extensively with other radiation professions, predominantly medical physicists, dosimetrists, and radiation therapists. In a multidisciplinary context, there is also significant interaction with other radiologic subspecialties (notably diagnostic radiology, interventional radiology, nuclear medicine). Importantly, ACGME program requirements require accredited departments to have dedicated faculty members with expertise in radiation and cancer biology and radiation physics.^25^

## 4.6 CURRENT STATUS AND FUTURE OUTLOOK

### 4.6.1 Radiology

**FIGURE 3:**
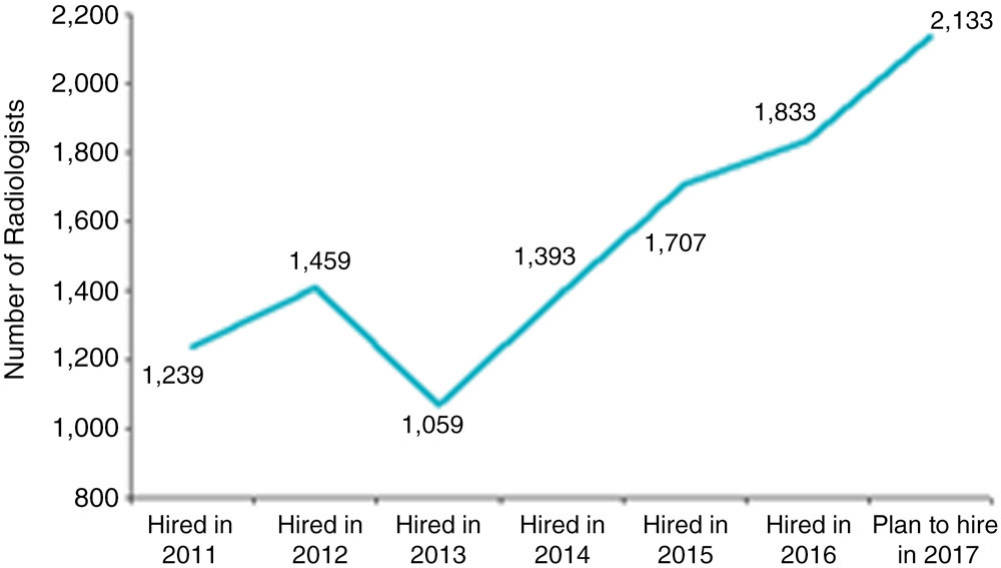
Actual and projected hiring trends for the years 2011–2017, as identified by the 2017 American College of Radiology (ACR) Workforce Survey.^12^ (Previously published and with permission from the JACR.)

From one perspective, the future outlook of the radiology workforce appears to be insufficient. The ACR Workforce survey^12^ results showed that a large number of radiologists are over the age of 55 and working full‐time; therefore, a significant workforce shortage could occur as this large group of radiologists retires. Indeed, in 2017, data suggested that between 1800 and 2400 positions would be opened, with only approximately 1200 individuals finishing fellowships (Figure 3). With the additional issue of burnout becoming more significant among radiologists than in the past, the possibility of increased retirement rates among senior radiologists is of real concern and will need to be monitored carefully.^31^ Additionally, there is an increasing number of radiologists who prefer to work part‐time. Since a statistically significant higher number of female radiologists choose to work part‐time relative to their male colleagues, efforts to increase gender diversity may exacerbate a shortage of trained radiologists. However, countering this trend is the development and potential adoption of machine learning and artificial intelligence (AI), with new computer‐aided diagnostic tools assisting radiologists and others in the interpretation of imaging examinations. AI could significantly alter the workflow and interpretative processes used by radiologists, thereby leading to a decrease in the number of radiology professionals needed to handle the workload.^32^, ^33^, ^34^ Therefore, it is currently unclear whether AI will be a disruptive technology to the radiology workforce or simply a means of balancing the trend toward part‐time work and the growing number of retirements. Although as a result of the Covid pandemic, there has not been a recent ACR Workforce Survey, nonetheless it does appear that there is currently a significant shortage of diagnostic radiologists subspecializing in chest and body imaging, including ultrasound and pediatrics.

#### 4.6.1.1 Interventional radiology

The outlook for the interventional radiology workforce is promising. The recent change in certification and training emphasizes the key role of interventional radiologists in the evaluation and management of patients with conditions amenable to image‐guided therapies. Currently, the 250 VIR fellowship positions are consistently filled, and there is tremendous interest by medical students in this specialty, as evidenced by the highly competitive NRMP match in 2017 with over 300 applicants for 122 positions. The job market for interventional radiologists in practice appears strong, and in recent years, this has been one of the subspecialties of diagnostic radiology in greatest demand.^35^ Data from 2010 suggested a cumulative annual growth rate of approximately 15%.^36^

#### 4.6.1.2 Nuclear radiology and nuclear medicine

Various organizations, notably the ABNM, the ABR, the ACR, and the SNMMI, have long been concerned with the status of nuclear physician experts already in practice, as well as those entering the nuclear radiology and nuclear medicine fields.^37^, ^38^, ^39^ Comparing the 2016–2017 and 2010–2011 academic years, the relatively larger number of diagnostic radiology residency programs increased from 187 to 192, whereas the much small number of nuclear radiology fellowship programs decreased from 19 to 18, with few trainees enrolled in the latter years. The number of nuclear medicine residency programs decreased from 54 to 42, with a precipitous fall in the number of trainees, from 155 down to 82.^40^ Thus, the pipeline into nuclear medicine is clearly threatened. This may be the result of a loss of career opportunities,^41^, ^42^, ^43^ with many workplaces appearing to hire nuclear radiologists who are competent in other aspects of diagnostic radiology. Since nuclear radiology is incorporated into the daily clinical practice of many diagnostic radiologists who have completed the requisite training and achieved specialty board certification, it would follow that there are sufficient numbers of diagnostic radiologists to fill vacancies and meet overall needs. However, it can be posited that nuclear radiologists or specialists in nuclear medicine practice at higher levels, leading to better access, optimal utilization of technology and enhanced quality of patient care as a result. Given that the numbers of nuclear radiologists and nuclear medicine physicians are much lower than diagnostic radiologists, and that they are likely distributed in urban or large suburban settings, there might be well be a worsening shortage in these subspecialties, limiting access to the technologies and lowering the quality of care. Indeed, one of the major challenges to the future of nuclear radiology is the many other attractive competing subspecialties in diagnostic radiology, many of which have fellowship programs

### 4.6.2 Radiation oncology

The question of adequacy in the current balance between supply of radiation oncologists and demand for their services is difficult to answer definitively; however, recent graduating residents have indicated concerns over an increasingly competitive job market,^44^, ^45^ as noted in the 2017 ASTRO workforce survey.^18^ This perceived difficulty of finding a satisfactory job may be at least partially attributable to the desire to practice in a narrow range of geographies in a field that already struggles with geographic maldistribution^46^, ^47^ rather than a true global oversupply of physicians. A more complicated question is whether the number of incoming and practicing radiation oncologists is on a par with anticipated changes in demand. Extrapolating cancer incidence rates from Surveillance, Epidemiology, and End Results (SEER)^48^ onto the aging demographic projected by the U.S. Census Bureau^49^ predicts an approximately 25% increase in overall cancer incidence between 2015 and 2025,^50^ with an approximately 20% increase in radiotherapy demand between 2015 and 2025.^51^ In contrast, a separate study combining a claims‐based analysis of radiation oncology utilization with modeled projections of cancer prevalence based on SEER data estimated a 40% increase in demand for oncology services between 2012 and 2025.^52^ Coupled with the predicted 25% increase in radiation oncology FTEs over a similar time period, the former set of projections suggest supply will slightly outpace demand, while the latter study predicts a significant undersupply of radiation oncologists. Recognizing the importance of this issue, ASTRO has created a Radiation Oncology Workforce Task Force, which has led to the selection of an independent firm to execute an analysis of the future requirements of the radiation oncology workforce (www.astro.org/Blog/March‐2022/A‐Look‐Ahead‐at‐the‐Radiation‐Oncology‐Workforce‐i).

Prediction of demand for radiotherapy services in the United States is challenging since changes in practice also need to be considered. Practice trends, such as use of hypofractionation, re‐irradiation, increased observation for early stage prostate and breast cancers, stereotactic radiosurgery (SRS), SBRT, proton therapy, and brachytherapy, can all potentially change the demand for radiation oncology services. Other changes in treatment management, such as targeted therapies and immunotherapy, also have the potential to change the balance of utilization between radiation oncology and other oncologic treatment modalities. Alterations in reimbursement structure and insurance coverage also have the potential to impact which services will be reimbursed and, therefore, performed, affecting workforce needs.

## 4.7 SUMMARY AND RECOMMENDATIONS

For clarity, summaries are given separately for the specialties of radiology, nuclear radiology and nuclear medicine, and radiation oncology.

### 4.7.1 Radiology

To ensure that there is an adequate workforce, we must continue to make radiology an attractive field for medical students. We must work to reduce the workload and workplace issues contributing to burnout so that fewer radiologists feel the need to transition to an earlier partial or complete retirement than at present. We also need to create an environment where there is zero‐tolerance of bullying so that trainees feel safe.^53^, ^54^

We must develop appropriate expectations regarding the volume of work required, better solutions for providing after‐hours care, and the maintenance and restoration of respect and professional standing for radiologists in order to provide a pleasant, appealing and desirable work environment.^31^, ^55^

### 4.7.2 Nuclear radiology and nuclear medicine

Support should be provided to ongoing efforts to interest medical students and diagnostic radiology residents in nuclear radiology and nuclear medicine. For example, the ACR and the SNMMI are championing interest groups to inform and engage students and trainees early in their training. Some possible tactics are to create outreach programs that foster interest in a career in nuclear radiology since there is a clear job market. In addition, in order to ensure a steady pipeline of skilled and employable clinical practitioners, educators, and researchers, innovative training pathways may need to be created. Such pathways exist and do, in fact, produce graduates who are grounded in contemporary diagnostic radiology and are prepared for an integrated practice. Potential solutions are to introduce nuclear radiology and nuclear medicine early in diagnostic radiology residency and to offer more innovative 4‐year and 5‐year training programs.

### 4.7.3 Radiation oncology

The most recent study projecting an oversupply of radiation oncologists^18^ led to discourse about strategies aimed at maintaining an adequate workforce.^56^, ^57^, ^58^ Proposed solutions included:

1. *Market‐based solution*: In this strategy, it would be anticipated that medical students self‐regulate according to perceptions about job availability and desirability. However, examples of supply and demand imbalance from international radiation oncology job markets^59^, ^60^ and domestic markets in other specialties, such as pathology^61^ and diagnostic radiology,^62^ call into question the adequacy of a purely market‐based approach, especially since there is a substantial temporal separation between entry into training and job‐seeking.
2. *Centralized control of residency size*: While no single organization is currently tasked with assessing radiation oncologist supply and demand, candidate organizations include ASTRO, the ACGME, ADROP, and the Society of Chairs of Academic Radiation Oncology Programs. There is historical precedent for such a centralized approach since residency curricula were lengthened from 3 to 4 years and training positions reduced from 137 to 93 in the face of decreasing job opportunities in the 1990s.^63^ However, there are antitrust concerns about leveraging this approach in the future.^57^
3. *Alternative residency curriculum*: Analogous to the current residency training pathway that allows for a more research‐based focus,^28^ alternative residency curricula could be developed if there are concerns about physician shortages. These could include a shorter 3‐year clinical track to address global undersupply or incentives such as loan repayment to encourage correction of geographic maldistribution.^47^, ^64^, ^65^ At present, radiation oncology residents are required to have training in cancer and radiation biology and radiation physics. As changes occur in radiation oncology patient management and workflow, consideration may also need to be given to incorporating training in genomics, immunology, artificial intelligence, and other areas of emerging importance

The recommendations below are consensus expert opinions on actions needed to ensure that the medical specialties considered will be able to meet the nation's future needs. The Committee intentionally declined to recommend detailed methods, timelines, responsibilities of individual organizations, and funding sources. These complex subjects are outside the scope of this Review, and indeed, the Committee was prohibited from activities that could be construed as advocacy.

### The authors recommend the following items to ensure the future adequacy of the nation's professional medical workforce

1. Professional organizations, such as the ACR, ASTRO, and SNMMI, should be encouraged to undertake annual workforce surveys covering all of the radiation medical professions; these would generate a better real‐time understanding of workforce trends. Surveys should include the monitoring of technological advances, such as artificial intelligence and deep learning, to determine how these are affecting the workforce. In addition, professional organizations, such as ACR, ASTRO, RSNA, AUR, APDR, and SNMMI, should be encouraged to develop a new national group, committee or commission, which can use survey data to make national recommendations or decisions regarding the appropriate number of trainees in all of the radiation medical professions discussed in this chapter.
2. There should be a concerted effort to stimulate interest in nuclear radiology and nuclear medicine, subspecialties in diagnostic radiology and radiation oncology, by reaching out to medical students (and diagnostic radiology residents), providing evidence of the relevance of these fields at multidisciplinary conferences and within medical departments. Lobbying efforts should consider the inclusion of a dedicated course on radiation within the first 2 years of medical study. Interested parties that could be involved in such an effort include organizations such as the ACR, ADROP, APDR, AAMC, AUR, RSNA, and SNMMI.
3. Several medical specialties, such as general practitioners, are encouraged to work in underserved geographic areas through offers of medical school debt forgiveness, etc. Organizations such as the ACR and American Medical Association should be urged to work with states to extend these approaches to specialties with projected shortages, such as interventional and nuclear radiology.
4. The medical industry, including the radiation sector, should continue to lead new initiatives to increase productivity, while maintaining world‐leading standards for quality and safety of care. They should work to develop strategies and resources to improve the efficient and effective use of physician time.

## AUTHOR CONTRIBUTIONS

All the authors listed have contributed directly to the intellectual content of the manuscript.

## CONFLICT OF INTEREST

The authors declare that there is no conflict of interest that could be perceived as prejudicing the impartiality of the research reported.

## Data Availability

Data sharing is not applicable to this article as no datasets were generated or analyzed during the current study.
